# A gallery of the key characters to ease identification of *Dermanyssus gallinae* (Acari: Gamasida: Dermanyssidae) and allow differentiation from *Ornithonyssus sylviarum* (Acari: Gamasida: Macronyssidae)

**DOI:** 10.1186/1756-3305-5-104

**Published:** 2012-05-30

**Authors:** Antonella Di Palma, Annunziata Giangaspero, Maria Assunta Cafiero, Giacinto S Germinara

**Affiliations:** 1 Dipartimento di Scienze Agroambientali Chimica e Difesa Vegetale (DiSACD), University of Foggia, Via Napoli 25, 71100, Foggia, Italy; 2 Dipartimento di Scienze delle Produzioni e dell'Innovazione nei Sistemi Agro-alimentari Mediterranei (PRIME), University of Foggia, Via Napoli 25, 71100, Foggia, Italy; 3 Istituto Zooprofilattico Sperimentale della Puglia e della Basilicata, Via Manfredonia 20, 71100, Foggia, Italy

**Keywords:** Poultry red mite, Northern fowl mite, Scanning electron microscopy, Light microscopy, External morphology, Identification

## Abstract

**Background:**

*Dermanyssus gallinae* (poultry red mite) is a major threat for the poultry industry and is of significant interest for public health. Identification of *D. gallinae* can be difficult for scientists not familiar with mite morphology and terminology especially when trying to use identification keys. Moreover, this species may easily be confused with another dermanyssoid mite, *Ornithonyssus sylviarum* (northern fowl mite), which often shares the same hosts and environment.

**Methods:**

Specimens of *D. gallinae* were collected at poultry farms in the Puglia and performed for light and scanning electron microscopy observations, identification and micrographs. Moreover specimens of *O. sylviarum* were collected separately macerated and mounted on slides for light microscopy observations, identification and pictures.

**Results:**

The micrographs used in this study, based on LM and SEM observations, highlight the following important identifying characters of *D. gallinae*: the prominent shoulders of the dorsal shield and the jagged edges of the shield reticulations, the position of setae *j*1, *s*1 and the epigynal pores, and the presence on tibia IV *pl* of one seta. Additional micrographs highlighting the shape of the dorsal (abruptly narrowed posteriorly) and epigynal (narrowly rounded posteriorly) shields and the chelicera (elongate, with distinct digits) of *O. sylviarum* enable its differentiation from *D.gallinae*.

**Conclusion:**

The photographic support provided here (both LM and SEM pictures) can be considered a practical tool for scientists who are not well acquainted with the morphology of *D.gallinae*, and who are involved with classical and molecular systematics, veterinary and human health aspects of poultry red mites.

## Background

*Dermanyssus gallinae* (De Geer 1778) (poultry red mite) is a cosmopolitan hematophagous ectoparasitic mite of wild, domestic and synanthropic birds [1,2] and which may also feed upon mammalian hosts [3-6]. *D. gallinae* is a significant pest of poultry worldwide [7-9] and a serious economic threat mainly to the laying hen sector [10,11] in any farming system (cages, barns, free-range and organic farming), including the recently introduced “colony” system [12]. *D. gallinae* is responsible for stress behaviour in its poultry hosts, reduced egg production and egg grade, anaemia, and diminished disease resistance [11,13,14]. *D. gallinae* is also a vector of several infectious disease agents [15]. Though relatively rare, the scientific literature records episodes of hen mortality associated with *D. gallinae*, mainly involving chicks [16]. The poultry red mite is also widely reported as being responsible for dermatological problems of varying severity in humans, both in poultry workers (technicians, farmers, veterinarians) [1,17]) and in urban residents. In fact in the last case, military personnel living in barracks, hospital patients, and office employees may be exposed to mite-infested synanthropic birds [18-23].

As a consequence of its economic and sanitary importance, many researchers working in different fields (acarologists, veterinarians, biologists, physicians, dermatologists, parasitologists) are often obliged to identify specimens of *D. gallinae* based on morphological characters. Taxonomic keys to *D. gallinae* are available in the literature [24], but they usually do not include high resolution photographs. That is why, Moss’s key may not be a straightforward tool for scientists who may have an interest in parasitic mites but have little or no training in their morphology and identification. Moreover, identification of *D. gallinae* can be confounded by the presence of similar dermanyssoid parasites such as *Ornithonyssus sylviarum* (Canestrini and Fanzago, 1877), which may, at least in European Countries, share the same host species and environment.

For this reason it is felt that those involved with the broader systematic, molecular, health or economic aspects of *D. gallinae* might benefit from a gallery of light and scanning electron micrographs illustrating the characters used by Moss [24,25] to identify this species. Accordingly, we have photographed every *D. gallinae* feature mentioned in Moss’ keys and have labelled them to pinpoint their appearance and location. We have also included micrographs illustrating the most important morphological differences between *D. gallinae* and *O. sylviarium* in order to better differentiate these morphologically similar species.

The adult female is the only stage/sex described here and in other available keys, probably as discriminant morphological characters appear mainly in this stage. However, in addition to pictures of female *D. gallinae*, supporting Moss’s key, we also provide illustrations of other stages (larva, protonymph, deutonymph and male) both for completeness and to aid non-specialists in distinguishing females (to which the key can be applied) from other available stages.

Finally, for those not familiar with mite morphology a glossary is included to explain the terms used to identify the morphological structures commonly used in the identification keys ( Additional file 1: Table S1).

## Methods

From May to September 2009, mite samples were collected at poultry farms in the Puglia Region (Italy) during daylight hours, from a variety of sites, including beneath feed troughs, inside cage fittings and fastening clips, under egg conveyor belts, and under manure belts. Mites were collected directly with a fine brush and held in closed petri dishes. After collection, mites were placed into labelled plastic bags and taken to the laboratory where they were separated from dust and debris. Half of the 368 collected specimens were directly frozen at **-**20°C, and the remaining specimens were transferred into vials containing 70% ethanol. Frozen specimens were macerated in lactophenol for one week at 45°C on a hot plate, and then mounted on slides with Hoyer’s medium for light microscopy (LM) observations [26,27].

The maceration process assured that specimens were clear enough for light microscopy to allow unimpeded observation of cuticular structures at any plane of focus. Identification of females was performed following Moss’ keys [24,25].

Specimens stored in 70% ethanol were prepared for scanning electron microscope (SEM) photography. They were dehydrated through a graded ethanol series, dried using a Baltec CPD030 critical point dryer, mounted on SEM stubs using conductive carbon adhesive tabs and sputter coated with palladium-gold using a Baltec SCD005 coating apparatus. Specimens were observed and photographed with a Zeiss EVO40 XVP scanning electron microscope with a digital camera.

Specimens of *Ornithonyssus sylviarum* (Canestrini and Fanzago, 1877) were collected earlier (2000) from a white wagtail (*Motacilla alba*, Linnaeus 1758) nest, macerated in lactophenol for few days at 45°C on a hot plate and mounted on slides using Hoyer’s medium [26,27].

Observations, identification and light images were obtained using an Olympus BX51 with an Olympus E330 camera.

## Results

*D. gallinae* belongs to the parasitiformes order Mesostigmata (Gamasida), in the suborder Monogynaspida, cohort Gamasina, superfamily Dermanyssoidea, family Dermanyssidae [27]. Neopodospermy (=sperm transfer through accessory insemination pores located close to the legs) occurs in dermanyssoid mites, along with the morphological and functional adaptations related to podospermy. The male genital opening is presternal, and the chelicerae are modified as gonopodes and provided with a sperm transfer process (spermatodactyl) arising from the movable digit of the chelicerae. Females have a sperm access system for sperm reception, and probably for storage and capacitation [27-30].

**Figure 1:**
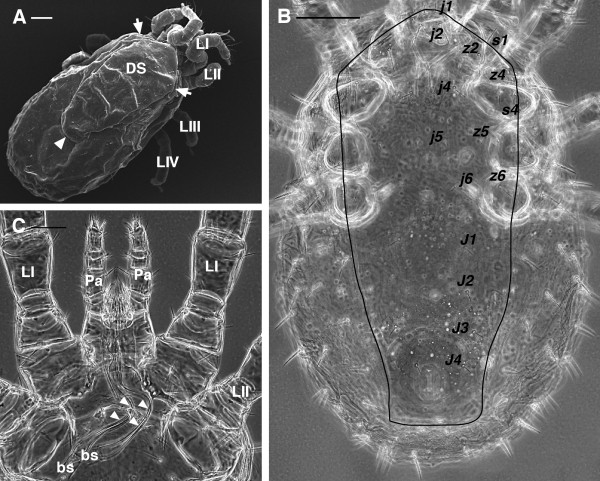
***Dermanyssus gallinae*females.(A)** SEM image: dorsal overview showing the idiosoma broadly rounded posteriorly, the single dorsal shield with prominent shoulder (arrows) and the truncate posterior margin (arrowhead). **(B)** LM image of the dorsal shield (outline traced), chaetotaxy according to Moss [24]. **(C)** LM picture of the anterior region of the body with the evident and elongate second cheliceral articles, far exceeding the basal segment in length (arrowheads). Abbr: bs, basal segment of chelicerae; DS, dorsal shield; LI-LIV, leg I, II, III, IV; Pa, pedipalp. Scale bar: 100 μm A, C; 50 μm B.

**Figure 2:**
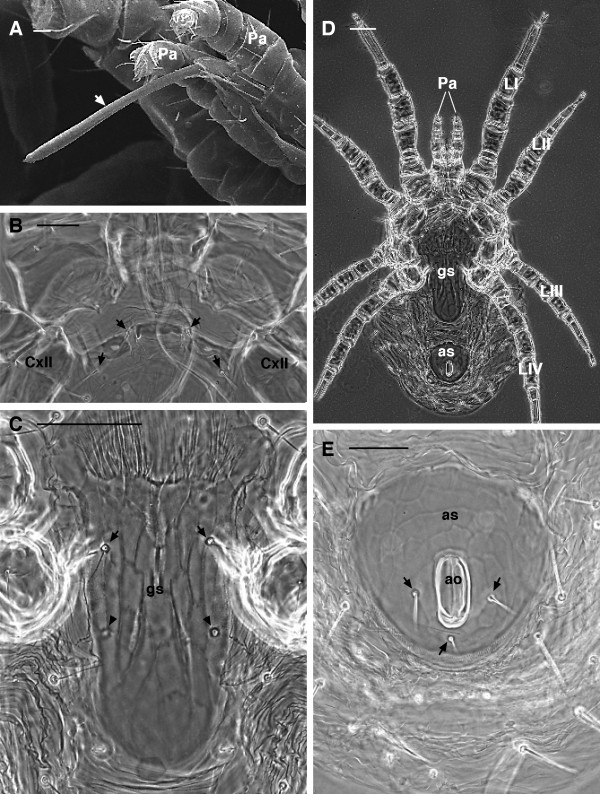
***Dermanyssus gallinae*females.(A)** SEM image: detail of the stylet-like second cheliceral article (arrow). **(B)** LM: detail of the wider than long sternal shield, bearing 2 pairs of sternal setae (arrows). **(C)** LM: detail of the genitoventral shield with 1 pair of setae (arrows) and 1 pair of epigynal pores (arrowheads). (**(D)** LM: overview of the ventral side. Note the genitoventral (epigynal) shield broadly rounded posteriorly. **(E)** LM: detail of the anal shield with three anal setae (arrows). Abbr: ao, anal opening; as, anal shield; CxII, coxa II; gs, genitoventral shield; LI-IV, leg I-IV; Pa, pedipalp. Scale bar: 10 μm A; 50 μm B, C, E; 100 μm D.

The Family Dermanyssidae is characterized as follows:

1. Idiosoma broadly rounded posteriorly (Figure 1A-B)

2. Second cheliceral article of female elongate, far exceeding the basal segment in length (Figure 1C, 2A)

**Figure 3:**
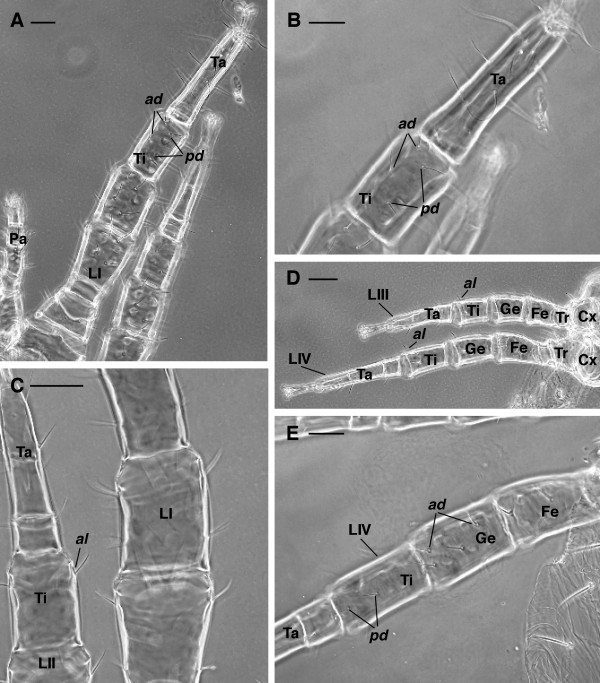
***Dermanyssus gallinae*females: dorsal view showing the dorsal shield chaetotaxy used in identification of the species.(A)** LM **(B)**, **(C)** SEM pictures. According to the key, seta *j*3 on the dorsal shield is missing (arrow point to the approximate position where this seta should be present) while *j*1 and *s*1 are located on the dorsal shield. Dorsal chaetotaxy according to Moss [24]. Scale bar: 50 μm A; 100 μm B; 10 μm C.

**Figure 4:**
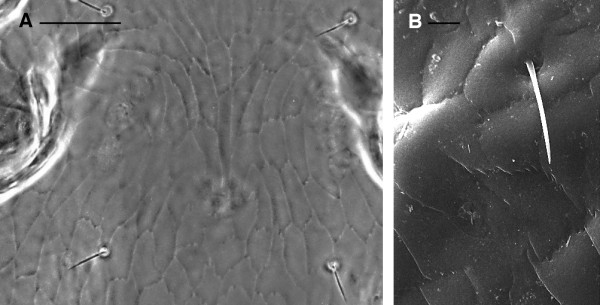
***Dermanyssus gallinae*females: leg chaetotaxy LM.(A)** tibia I with 2 anterodorsal and 2 posterodorsal setae. **(B)**, enlargement of (A). **(C)** tibia II with 1 anterolateral seta. **(D)** tibia III and tibia IV with 1 anterolateral seta. **(E)** genu IV with 2 anterodorsal setae and tibia IV with 2 posterodorsal. Abbr.: *ad*, anterodorsal; *al*, anterolateral; Cx, coxa; Fe, femur; Ge, genu; LI-IV, leg I-IV; Pa, pedipalp; *pd*, posterodorsal; Ta, tarsus; Ti, tibia; Tr, trochanter. Scale bar: 50 μm A, B, C, E; 100 μm D.

**Figure 5:**
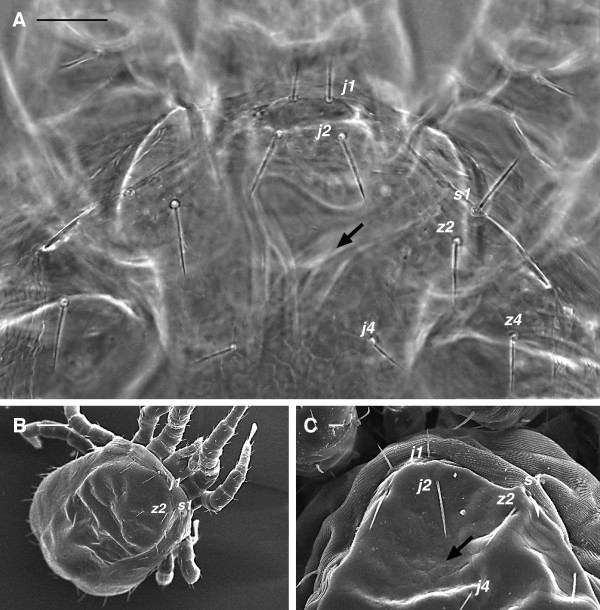
***Dermanyssus gallinae*females.** Leg IV chaetotaxy. **(A)**, **(B)**, LM, overview of the leg IV (A) and detail of the tibia with 1 posterolateral seta (B). **(C)** SEM picture showing the location of the setae on genu and tibia IV. Abbr: *ad*, anterodorsal; Cx, coxa; Fe, femur; Ge, genu; LIII-IV, leg III-IV; *pd*, posterodorsal; *pl*, posterolateral seta; Ta, tarsus; Ti, tibia; Tr, trochanter. Scale bar. 50 μm A, B; 40 μm C.

According to Moss [24] the genus *Dermanyssus* Dugés presents the following character states:

1. lack of seta *j*3 on the dorsal shield (Figures 1B, 3A-C)

2. sternal shield (the median ventral sclerite between leg II and III) narrowed, distinctly wider than long, bearing 1–2 pairs of sternal setae (Figure 2B)

3. tibia I *ad* (anterodorsal) and *pd* (posterodorsal) with two setae (Figures 4A-B)

4. tibia II-IV with 1 seta *al* (anterolateral) (Figure 4C-D)

5. genu IV *ad* (anterodorsal) and tibia IV *pd* (posterodorsal) each with 2 setae (Figures 4E, 5C).

Regarding the nomenclature of leg setae, each segment is considered to have four seta-bearing surfaces: dorsal, ventral, anterolateral and posterolateral. In particular, the anterior and posterior faces of the leg segments refer to the position adopted when the leg is extended laterally, more or less at right angles to the longitudinal axis of the body.

In general appearance, *D. gallinae* presents a single dorsal shield (Figure 1A-B) that tapers posteriorly and has a truncate posterior margin (Figure 1A-B). Chelicerae are long and styliform (Figures 1C, 2A). The sternal shield (median sclerite between leg II and III) has two pairs of setae (Figure 2B), and a third pair is located more posteriorly and distinctly separated from the others. The genitoventral shield is posteriorly rounded and bears one pair of seta (Figure 2C-D). The anal shield has three setae (Figure 2D-E).

Moss [24,25] noted that the most useful setae for differentiation of *D. gallinae* from other members of the genus are those in the “*j*” series of the dorsum (presence or absence, location on or off the dorsal shield). Leg setae also are useful for species identification.

**Figure 6:**
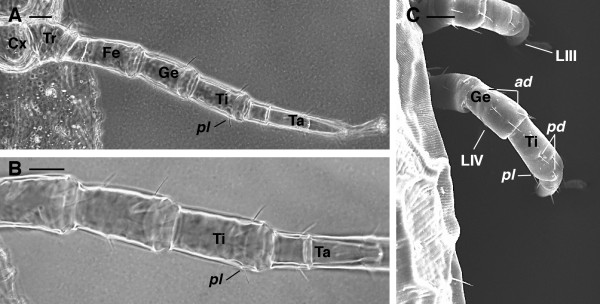
***Dermanyssus gallinae*females: detail of the dorsal shield reticulation with evident jagged edges.(A)** LM, **(B)** SEM pictures. Scale bar: 50 μm A; 10 μm B.

Therefore, following Moss’ keys, *D. gallinae* presents the following characters:

1. dorsal shield with prominent shoulder (Figure 1A-B)

2. shield reticulations with jagged edges (Figure 6A-B)

3. *j*1 always, and *s*1 usually on dorsal shield (Figures 1A-B, 3A-C)

4. epigynal pores on shield (Figure 2C)

5. tibia IV *pl* with 1 seta (Figure 5A-C)

**Figure 7:**
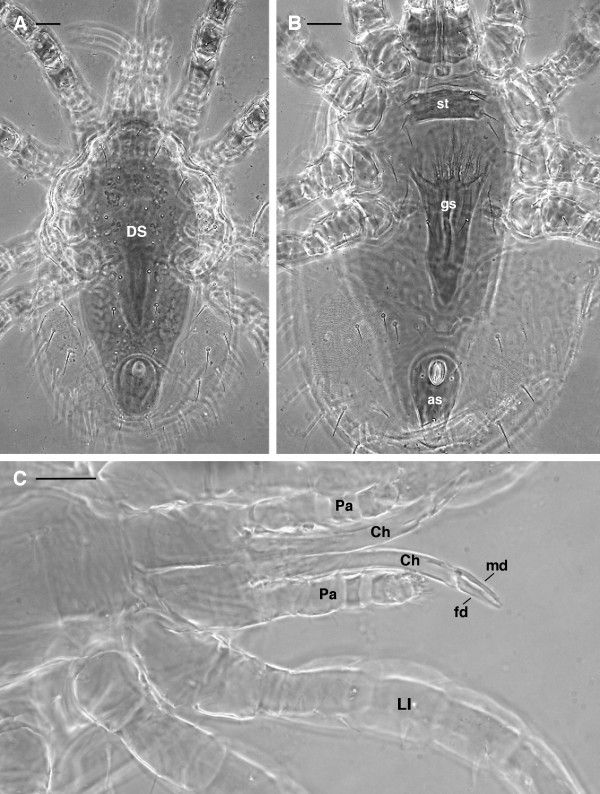
***Ornithonyssus silviarum *female: LM. (A)** dorsal view with a single dorsal shield narrowing posteriorly. **(B)** ventral view with the genitoventral (epigynal) shield attenuate or narrowly rounded posteriorly. **(C)** detail of the elongated chelicerae with well developed and distinct fixed and movable digit. Abbr: as, anal shield; Ch, chelicera, DS, dorsal shield, fd, fixed digit; gs, genitoventral shield; LI, leg I; md, movable digit; Pa, pedipalp; st, sternal shield. Scale bar: 50 μm.

*O. sylviarum* is also a dermanyssoid mite, but it is placed in the family Macronyssidae rather than the Dermanyssidae [27]. *O. sylviarum* may be distinguished from *D. gallinae* as follows:

1. the chelicerae of females are elongate, but with well developed and distinct fixed and movable digits (Figure 7C) (whip-like with no evident chela in *D. gallinae*) (Figures 1C, 2A)

2. the genitoventral (epigynal) shield is attenuate and narrowly rounded posteriorly (Figure 7B) (broadly rounded posteriorly in *D. gallinae*) (Figure 2C-D).

3. the dorsal shield of *O. sylviarum* is abruptly narrowed posteriorly (Figure 7A) (more smoothly narrowed in *D. gallinae*).

**Figure 8:**
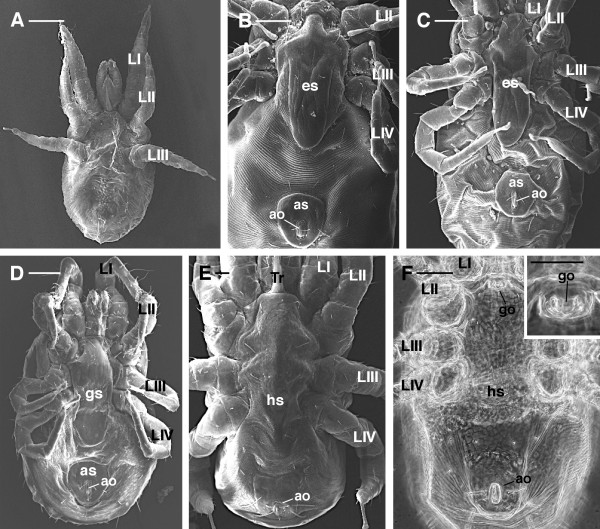
***Dermanyssus gallinae*: overview of the different stages.(A)** SEM, larva ventral view: it is evident that there are only three pairs of legs with little sclerotization and without indication of external genitalia; **(B)**, **(C)** SEM: ventral view of proto and deutonymph respectively: epigynal shield reduced compared to the female; **(D)** SEM ventral view of female with the genitoventral (epigynal) shield completely developed; **(E)** SEM, **(F)** LM, ventral view of the male showing the holoventral shield and a small genital opening set in a presternal position (F and inset). Abbr.: ao, anal opening; as, anal shield; es, epigynal shield; gs, genitoventral shield; go, genital opening; hs, holoventral shield; LI-IV, leg I-IV; Tr, tritosternum. Scale bar: 100 μm A-D, F and inset; 20 μm E.

Finally it is important to stress that in order to correctly use the key presented for *D. gallinae*, the user must be certain that the mite under observation is a female. Females may be easily distinguished from the other stages (larva, proto- and deutonymph, male) as follows:

· the larva is a hexapod form with little or no sclerotization and without indication of external genitalia (Figure 8A).

· nymphs are octopod as adults, but undergo progressive shield differentiation (compare Figure 8B and C) with each molt until adult stage. Thus, the epigynal shield in nymphs (Figure 8B-C) appears reduced compared to the female (Figure 8D), and there is no genital opening.

· males have a small presternal genital opening (Figure 8F), and the intercoxal region is covered by a sternogenital sclerite. Fusion of the sternogenital and ventroanal elements results in a holoventral shield (Figure 8E-F). Conversely, females have an epigynal shield (Figure 8D). The male chelicerae are modified as gonopods for sperm transfer and hence provided with a spermatodactyl.

## Discussion

The aim of this report is to provide researchers and practitioners with a gallery of light and scanning electron micrographs illustrating the characters used by Moss [24,25] to identify *D. gallinae*, whilst also distinguishing this species from *O. sylviarum*. Such an iconographic tool should greatly improve correct identification of this species by researchers and practitioners working with *D. gallinae*, particularly by individuals with otherwise limited experience in taxonomy. Correct identification of *D. gallinae* is critical if appropriate treatment of infested premises – both industrial and domestic – is to be recommended to both poultry producers and individuals affected by this mite. Historically this has proven to be a difficult task, with some host records of *D. gallinae* published before Evans and Till’s revision [31] probably incorrect because of the tendency to assume that all collections of *Dermanyssus* were *gallinae*. Though improvements to keys for *D. gallinae* have been made since [24,25], these have not been able to utilize high resolution digital imaging techniques and the open-access publishing model, as the current work does, to optimize usability.

Apparent confusion with similar mite species, primarily *O. sylviarum* which may cohabit with *D. gallinae* and pose similar problems for hen [7,9,32] and human [4,18,20-23,33] health, may also hamper positive identification and appropriate treatment of both species. Hence, the current work also makes comparison among these two mites for the benefit of the end user.

Where possible the authors recommend the use of the pictorial key presented in unison with that of Moss [24,25], on which the identification of *D. gallinae* used here is based, particularly as many characters listed may be variable (making it potentially useful to consult detailed illustrations [24,25] as well as digital imagery). For the same reasons we recommend examination of several mites per sampled population in order to achieve optimally reliable identification of *D. gallinae* using the key presented. Illustrations and descriptions of the male and immature stages given herein should help to distinguish these from females, which are the only suitable stage for morphological identification. Since *D. gallinae* usually occurs in huge colonies, collection of females for taxonomic use should not be limiting.

## Conclusions

We believe that this collection of key character micrographs (both LM and SEM pictures) will simplify identification of *D. gallinae* and aid in its differentiation from *O. sylviarum* for those who are involved with the broader systematic (classical and molecular), veterinary and human health aspects of poultry mite parasites, but who are not well acquainted with their morphology.

## Abbreviations

LM, light microscope; SEM, Scanning electron microscopy.

## Competing interests

The authors declare that they have no competing interests.

## Authors’ contributions

AD identified the specimens, performed them for electron and light microscopy and carried out the light and SEM observations, organized the photo gallery and drafted the manuscript. AG conceived the study, collected and identified the samples, and helped to draft the manuscript. MAC participated in the design of the study, collected and identified the samples and reviewed the manuscript. GSG helped to perform the samples and carry out light and SEM observations and reviewed the manuscript. All authors read and approved the final version of the manuscript.

## Supplementary Material

Additional file 1 **Table S1.** Glossary of the main morphological terms used in the key (listed in alphabetical order). Click here for file
