# Supplementary material for: A gallery of the key characters to ease identification of Dermanyssus gallinae (Acari: Gamasida: Dermanyssidae) and allow differentiation from Ornithonyssus sylviarum (Acari: Gamasida: Macronyssidae)
Source: Parasit Vectors. 2012 May 30;5:104. doi: 10.1186/1756-3305-5-104 (PMC3419681; doi:10.1186/1756-3305-5-104)
Supplement: Additional file 1 — Table S1. Glossary of the main morphological terms used in the key (listed in alphabetical order). [file 1756-3305-5-104-S1.doc]

Glossary of the main morphological terms used in the key (listed in alphabetical order):

- **Anal shield**: small sclerite, confined to the posterior part of the body on the ventral side. It is named after the cuticular structure that it bears (the anal opening).
- **Chelicerae**: first pair of appendages of the mouthparts composed of three articles: first (basal) article, second (middle) article bearing the fixed digit and the third article (the movable digit).
- **Dorsal shield**: a large sclerite dorsally located to cover the body.
- **Genitoventral (**epigynal**) shield**: posteriorly to the sternal shield, between legs IV, there is the flap like genital sclerite that can be enlarged posteriorly to coxae IV.
- **Gnathosoma**: anterior region of the body containing the mouthparts.
- **Idiosoma**: body region posterior to the gnathosoma composed of the podosoma (where legs are inserted) and opisthosoma (portion behind the legs).
- **Second cheliceral article**: second (middle) article that ends with the fixed digit.
- **Sternal shield**: the median ventral sclerite between legs II and III.
- **Tritosternum**: small, unpaired cuticle structure located between coxae I and presenting a base usually terminating in two elongated pilose processes.
